# Investigating the Effects of C1q‐Blocking Using a CDC‐Mutant 3D6 Antibody on ARIA in 5XE4 Mice

**DOI:** 10.1002/alz70861_108832

**Published:** 2025-12-23

**Authors:** Brianna R Colletti, Praveen Bathini, Ilina Navani, David M. Holtzman, Fareeha Saadi, Stephan Schilling, Jens‐Ulrich Rahfeld, Cynthia A Lemere

**Affiliations:** ^1^ Ann Romney Center for Neurologic Diseases, Brigham and Women's Hospital, Boston, MA USA; ^2^ Maastricht University, Maastricht Netherlands; ^3^ Harvard Medical School, Boston, MA USA; ^4^ Washington University School of Medicine, St. Louis, MO USA; ^5^ Fraunhofer Institute for Cell Therapy and Immunology, Halle Germany; ^6^ Brigham and Women's Hospital, Harvard Medical School, Boston, MA USA

## Abstract

**Background:**

Anti‐amyloid immunotherapy is associated with amyloid related imaging abnormalities (ARIA), an adverse vascular side effect that impacts ∼12‐40% of AD patients. Our lab previously identified a link between anti‐amyloid immunotherapy and complement activation in ARIA, particularly involving increased early colocalization of C1q with antibodies bound to CAA. This pilot study asked whether blocking C1q on the murine anti‐amyloid antibody 3D6 – known to induce ARIA ‐ would protect against ARIA.

**Method:**

Female 5XE4 mice (male 5xFAD lineage line 7031; Holtzman lab on a humanized floxed APOE4 knock‐in background) were immunized intraperitoneally weekly at 12.5 months‐old with either 15 mg/kg of 3D6 (n = 5) or CDC‐mutant (K322A) 3D6‐k (n = 4) over 9 weeks. Due to limited mouse availability, no isotype controls were included. Brain sections were stained with AmyloGlo (fibrillar amyloid), Prussian blue (microhemorrhages), H&E (RBC extravasation), anti‐murine IgG2a (surrogate for 3D6 and 3D6‐k), C3 and C1q/IgG2a (plaque colocalization). Complement gene expression was assessed via qPCR.

**Result:**

C1q/IgG2a colocalization in plaques was significantly lower in 3D6‐k treated mice compared to 3D6 treated mice (*p* =0.0001). Strong trends for lower *C3* (*p* =0.0842), *C3aR1* (*p* =0.0635), and *C4b* (*p* =0.0848) mRNA levels were observed in 3D6‐k treated mice. Fibrillar amyloid deposition was similar between groups indicating that the CDC mutation did not affect amyloid clearance compared to 3D6. CAA‐associated microhemorrhages and total microhemorrhages were non‐significantly lower by 80% and 57%, respectively, in 3D6‐k treated vs. 3D6 treated mice. RBC extravasation was lower by 77% (*p* =0.0672) and iC3b levels were lower by 36% (n.s.) in the 3D6‐k group compared to the 3D6 group.

**Conclusion:**

Immunization with 3D6‐k led to significantly less C1q/antibody colocalization in plaques compared to 3D6 immunization. Reductions in complement gene expression, microhemorrhage deposition, and RBC extravasation did not reach statistical significance due to small sample size, however, our preliminary data suggests that blocking C1q‐binding on 3D6 may reduce the incidence of ARIA and further supports our hypothesis that early complement activation upon anti‐amyloid antibody binding to CAA contributes to ARIA. Further studies with larger numbers of mice are underway to validate our results. Funding: NIH NINDS 1R01NS136122 and Cure Alzheimer’s Fund (CAL).

